# Proteogenomic characterization of ferroptosis regulators reveals therapeutic potential in glioblastoma

**DOI:** 10.1186/s12885-023-10894-3

**Published:** 2023-05-08

**Authors:** Xinzhuang Wang, Hong Zhang, Mingchu Zhang, Xuezhi Zhang, Wenbin Mao, Ming Gao

**Affiliations:** 1 Department of Neurosurgery, The First Affiliated Hospital of Zhengzhou University, Zhengzhou, China; 2 Department of Hematology, Liaocheng People’s Hospital, Liaocheng, China; 3 Department of Neurosurgery, First Affiliated Hospital of Harbin Medical University, Harbin, China

## Abstract

**Background:**

Ferroptosis is iron-dependent non-apoptotic cell death, that is characterized by the excessive accumulation of lipid peroxides. Ferroptosis-inducing therapy also shows promise in the treatment of cancers. However, ferroptosis-inducing therapy for glioblastoma multiforme (GBM) is still in the exploratory stage.

**Methods:**

We identified the differentially expressed ferroptosis regulators using Mann–Whitney U test in the proteome data from Clinical Proteomic Tumor Analysis Consortium (CPTAC). We next analyzed the effect of mutation on protein abundance. A multivariate Cox model was constructed to identify the prognostic signature.

**Results:**

In this study, we systemically portrayed the proteogenomic landscape of ferroptosis regulators in GBM. We observed that some mutation-specific ferroptosis regulators, such as down-regulated ACSL4 in EGFR-mutated patients and up-regulated FADS2 in IDH1-mutated patients, were linked to the inhibited ferroptosis activity in GBM. To interrogate the valuable treatment targets, we performed the survival analysis and identified five ferroptosis regulators (ACSL3, HSPB1, ELAVL1, IL33, and GPX4) as the prognostic biomarkers. We also validated their efficiency in external validation cohorts. Notably, we found overexpressed protein and phosphorylation abundances of HSPB1 were poor prognosis markers for overall survival of GBM to inhibit ferroptosis activity. Alternatively, HSPB1 showed a significant association with macrophage infiltration levels. Macrophage-secreted SPP1 could be a potential activator for HSPB1 in glioma cells. Finally, we recognized that ipatasertib, a novel pan-Akt inhibitor, could be a potential drug for suppressing HSPB1 phosphorylation, inducing ferroptosis of glioma cells.

**Conclusion:**

In summary, our study characterized the proteogenomic landscape of ferroptosis regulators and identified that HSPB1 could be a candidate target for ferroptosis-inducing therapy strategy for GBM.

**Supplementary Information:**

The online version contains supplementary material available at 10.1186/s12885-023-10894-3.

## Introduction

Glioblastoma multiforme (GBM), the most aggressive form of diffuse glioma (WHO grade IV), exhibits highly invasive characteristics with a poor prognosis [1]. Despite surgery and radiochemotherapy, cancer inevitably recurs and results in patient death [2]. The rapid development of therapy strategy provides more opportunities to fight against cancer, however, the selection of effective treatment is limited by a lack of biomarkers. The Cancer Genome Atlas (TCGA) [3] and Chinese Glioma Genome Atlas (CCGA) [4] have also demonstrated multiple potential markers at genome and transcriptome levels. For example, detection of serum microRNAs miR-17-5p, miR-125b, and miR-221 could contribute to predicting prognosis and response to a treatment strategy for GBM patients [5]. Nevertheless, characterization of ferroptosis regulators at protein levels is still the tip of the iceberg.

Ferroptosis is a new type of programmed cell death caused by an accumulation of toxic lipid peroxides [6–8]. Recent evidence has reported that ferroptosis is commonly dysregulated and contributes to tumorigenesis. In GBM, a previous study showed the contribution of enzymes ACSL4 and ACSL6 that activate polyunsaturated fatty acids (PUFA) to the phospholipid pool and the connection of PUFA-containing phosphatidylethanolamine to ferroptosis [9–11]. Besides, several researchers have suggested that ferroptosis is a promising treatment approach to cancer because of the high iron levels in cancer cells and their sensitivity to ferroptosis induction [12, 13]. However, exploration of ferroptosis-related therapeutic targets is still limited in GBM.

In this study, we characterized the proteogenomic landscape of ferroptosis regulators in human GBM. We found decreased acyl-CoA synthetase long-chain family member 4 (ACSL4) and increased fatty-acid desaturase 2 (FADS2) in EGFR-mutated and IDH1-mutated patients, respectively, which were associated with inhibition of ferroptosis in GBM. Furthermore, we identified the prognosis-related ferroptosis protein markers, in which heat shock protein beta-1 (HSPB1) and its phosphorylation were associated with the high-risk GBM patients. Finally, we also found a potential drug ipatasertib could inhibit HSPB1 kinases and might increase GBM ferroptosis activity.

## Materials and methods

### Data acquisition and preprocessing

Tandem Mass Tag (TMT)-based quantitative proteomics data of 99 GBM tumor tissues and 10 normal GTEx brain samples was from Wang et al. previous study [9]. Corresponding genome, transcriptome, and phosphoproteome were downloaded from Clinical Proteomic Tumor Analysis Consortium (CPTAC) [14]. For protein and phosphosite data, we removed the proteins/phosphosites with a missing rate of over 50%. The remaining missing values of proteins/phosphosites were imputed using the DreamAI algorithm [15]. The processed CPTAC proteomics data, and mutation data of corresponding samples were downloaded from the database LinkedOmics [16] (http://linkedomics.org/data_download/CPTAC-GBM/). The processed TCGA transcriptome data and mutation data were obtained from Genomic Data Commons Data Portal (https://portal.gdc.cancer.gov/). Ferroptosis-related genes were collected from FerrDb database and recently published papers [6, 12, 17–19]. Alternatively, transcriptome data from TCGA and CCGA (http://www.cgga.org.cn/) were employed for the validation cohorts.

### Differential expression analysis

We calculated the fold change (FC) of ferroptosis regulators between GBM tumor and normal samples. Then, we employed Mann–Whitney U test to test the protein abundances of ferroptosis regulators between GBM tumor and normal samples. *P*-value was adjusted by the Benjamini-Hochberg method. A total of 142 ferroptosis regulators with adjusted *P*-value < 0.01 were considered as the differentially expressed proteins.

### Construction of prognostic model based on ferroptosis regulators

We first trained the univariate Cox regression model based on the protein abundance of ferroptosis regulators. A total of 15 ferroptosis regulators with *P*-value < 0.05 were identified as the candidate prognosis-related regulators. Then, we divided the GBM-proteomics cohort into three parts, where two as the training set and one as the test set. In the training set, we constructed the multivariate Cox model using prognosis-related regulators. Subsequently, the bi-directional stepwise regression based on AIC (Akaike information criterion) value was utilized to select the ones that minimize AIC to attain the best model fit. Five ferroptosis regulators (HSPB1, GPX4, ACSL3, IL33, and ELAVL1) were identified as the ferroptosis prognostic signature (FPS), which showed a significant correlation with tumor samples’ overall survival (OS) probability. We also calculated the risk score based on the final stepwise Cox regression model for each patient, i.e.,

$$Risk\,score = \sum _{i = 1}^{n = 5}coe{f_i}*protei{n_i}$$

The risk scoring model could be expressed as Risk score = (-0.003987172 * ELAVL1) + (0.345390218 * GPX4) + (0.190379543*HSPB1) + (-0.318621832*ACSL3) + (-0.175877778 * IL33). A k-means algorithm was used with means of three highest and lowest values of risk score as initial centers for high-risk and low-risk groups. We also validated the efficiency of our risk model in the corresponding RNA-seq dataset and two external cohorts including the TCGA-GBM RNA-seq cohort and CCGA-GBM RNA-seq cohort.

### Phosphoproteome analysis

We analyzed the phosphorylation levels of the five prognostic ferroptosis regulators (HSPB1, GPX4, ACSL3, IL33, and ELAVL1) and identified 17 phosphosites in CPTAC phosphorylation data, including modifications on HSPB1 (HSPB1-S176s, HSPB1-S187s, HSPB1-S158s, HSPB1-S78sS82s, HSPB1-S199s, HSPB1-S50s, HSPB1-S82s, HSPB1-S83sS86s, HSPB1-S9s, HSPB1-S15s, HSPB1-T143t, HSPB1-S65s, HSPB1-Y133y, HSPB1-S86s, HSPB1-S98s), ACSL3 (ACSL3-S683s), and ELAVL1 (ELAVL1-S202s). Using univariate Cox regression analysis, we found that three out of the 17 phosphosites, all modified on HSPB1, were significantly associated with patients’ overall survival probability. We hence analyzed the HSPB1-related kinases supported by previous experimental evidence from the DEPOD database [20]. Kinases AKT1, MAPK14, MAPKAPK2, MAPKAPK3, PKD1, PRKACA, PRKD1, PRKG1, RPS6KB2, p70S6Kb were tested for the associations between all sufficiently detected phosphosites on the substrate. We proposed substrate phosphosite abundance (${Sp}_{i}$) depends on kinase protein (${K}_{j}$) and phosphosite (${Kp}_{j}$) expression, and substrate protein abundance (${S}_{i}$). Then the regression model was constructed as:

$${Sp}_{i} \tilde {K}_{j}+{Kp}_{j}+ {S}_{i}$$

*P*-values were adjusted for multiple testing using the Benjamini-Hochberg procedure.

### Identification of co-expressed proteins of HSPB1

We performed Spearman’s correlation analysis between HSPB1 abundance and other proteins. P-values were adjusted by the BH method. Proteins with Spearman’s coefficient > 0.6 and adjusted P-value < 0.01 were identified as the co-expressed proteins of HSPB1. Subsequently, we inferred the potential biological functions of HSPB1 based on its co-expressed proteins using Metascape [21].

### Tumor immune subtypes of GBM

To evaluate tumor immune subtypes, we applied the xCell algorithm to calculate the immune cell enrichment score (ES) [22]. We kept the cell types from the previous study, including B cells, CD4 + T-cells, CD8 + T-cells, DC, eosinophils, macrophages, monocytes, mast cells, neutrophils, NK cells, and microglia. Subsequently, we applied non-negative matrix factorization (NMF) to identify GBM TME subtypes using the NMF R-package [23]. Since NMF requires a non-negative input matrix we converted the ESs in the data matrix into a non-negative matrix based on the strategy from Wang et al. [9] as follows: (1) Matrix-1 was created by making all negative numbers zeroed; (2) Matrix-2 was created by making all positive numbers zeroed and taking the absolute values of all negative numbers; (3) Concatenate Matrix-1 and Matrix-2 resulting in a data matrix with positive values only and zeros and hence appropriate for NMF. The analysis resulted in three immune subtypes (IM1, IM2, and IM3).

### Association of potential drugs with risk ferroptosis regulators

We analyzed 52 glioma cell lines from the Genomics of Drug Sensitivity in Cancer (GDSC) project [24]. First, we sub-grouped these cell lines into high- and low-expression groups based on the median expression levels of five risk ferroptosis regulators, respectively. We continued to evaluate differences in drug response between cell lines in high- and low-expression groups using Wilcoxon rank-sum test. *P*-values were adjusted by Benjamini and Hochberg procedure.

## Results

### Dysregulated ferroptosis regulators in GBM

**Fig. 1:**
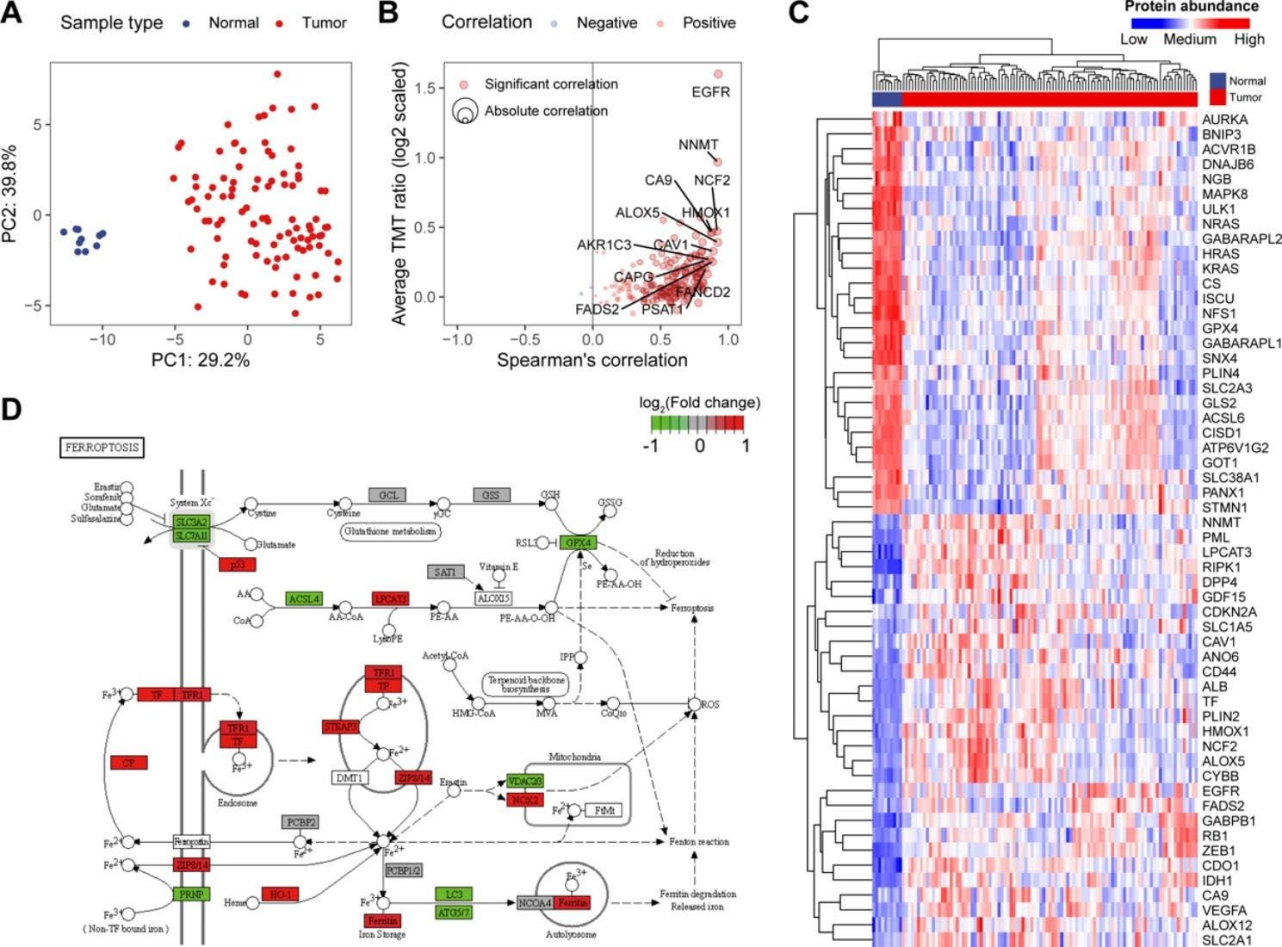
Overview of ferroptosis regulators in human GBM. (**A**) Unsupervised principal components analysis (PCA) of protein levels of ferroptosis regulators. (**B**) Correlations mRNA and protein levels of ferroptosis regulators. The top 10 ferroptosis regulators were labeled. (**C**) Heatmap showing protein abundances of ferroptosis regulators (|log2FC| > 1 and adjusted *P*-value < 0.01) between GBM tumor tissues and normal brain tissues. (**D**) Differentially expressed ferroptosis regulators in KEGG ferroptosis pathway. Pathway visualization using the R package “Pathview”

To characterize the dysregulated ferroptosis protein expression in GBM, we employed the CPTAC-patient cohort from Wang et al. based on high-throughput TMT mass spectrometry [9]. A total of 185 ferroptosis regulators were detected. There was a distinct difference between GBM tumor and normal brain samples based on ferroptosis regulators (Fig. 1A). We next performed Spearman’s correlation analysis between ferroptosis mRNA and protein abundances. Overall, the mRNA–protein correlation for the majority of ferroptosis regulators with outlier expression was high, implying the transcription regulation of ferroptosis regulators (Fig. 1B). We hence identified the differentially expressed ferroptosis regulators between GBM tumor and normal brain tissues using the Mann–Whitney U test (Fig. 1C). A total of 142 differentially expressed ferroptosis proteins were identified (adjusted *P*-value < 0.01). We also examined their transcriptional differences in an independent TCGA-GBM cohort. We found that about 64.1% of the ferroptosis regulators exhibited the same variation trend between normal brain and GBM tissues (Figure S1). Compared to the normal samples, some dysregulated ferroptosis regulators were associated with inhibited ferroptosis activity in GBM tissues (Fig. 1D) [25, 26], such as downregulated ACSL4 and upregulated HSPB1, corroborating previous observations [27–29].

### Proteogenomic landscape of ferroptosis regulators in GBM

**Fig. 2:**
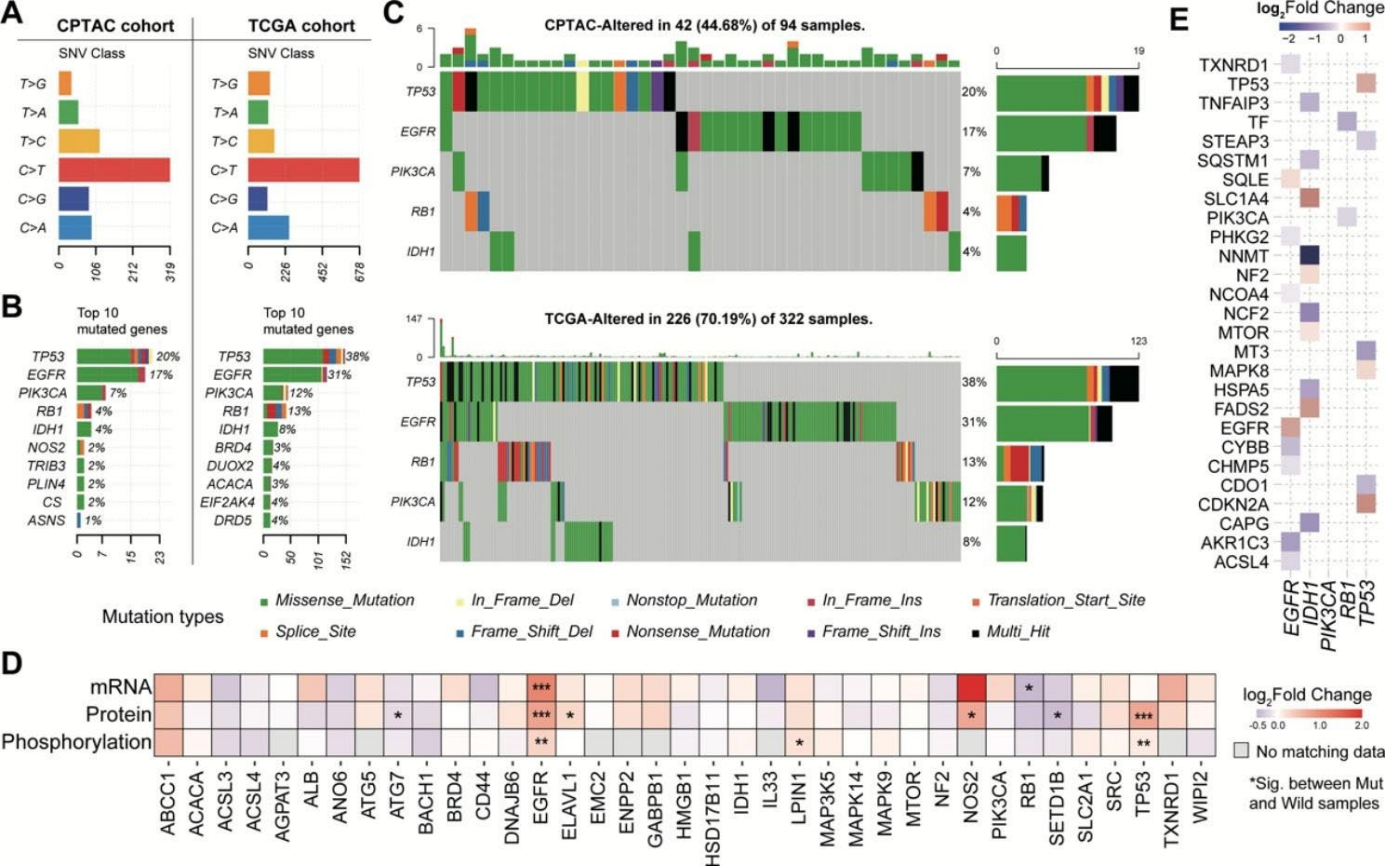
Proteogenomic regulation of ferroptosis regulators. (**A**) Barplot showing the SNV class in CPTAC-GBM and TCGA-GBM cohorts, respectively. (**B**) Top 10 mutated genes in CPTAC-GBM and TCGA-GBM cohorts, respectively. (**C**) Oncoplots showing the five common mutated genes in both CPTAC-GBM and TCGA-GBM cohorts. (**D**) Heatmap showing the differential expression of mRNA, protein, and phosphoprotein between mutated and wild samples. (**E**) Heatmap showing the effect of EGFR, IDH1, PIK3CA, RB1, and TP53 mutations on protein abundances of ferroptosis regulators

We next explored the effect of somatic mutation on the expression of ferroptosis regulators. In both TCGA and CPTAC cohorts, we observed that the mutation type from C to T (C > T) accounted for the highest proportion of ferroptosis-regulator mutations (Fig. 2A). Additionally, tumor protein p53 (TP53), epidermal growth factor receptor (EGFR), phosphatidylinositol-4,5-bisphosphate 3-kinase catalytic subunit alpha (PIK3CA), RB transcriptional corepressor 1 (RB1), and isocitrate dehydrogenase 1 (IDH1) had the highest mutation frequency in both datasets (Fig. 2B-C). Next, we investigated the influence of mutation on mRNA, protein, and phosphorylation abundances of ferroptosis regulators (Fig. 2D). We found that EGFR and TP53 mutation significantly increased their protein and phosphorylation abundances, implying they could play an important role in the ferroptosis of GBM. Notably, previous studies have demonstrated that deprivation of cystine led to increased cell death, generation of reactive oxygen species (ROS), and synchronous ferroptosis in cells expressing an activated EGFR mutant [30]. Additionally, treatment of xenografts derived from EGFR mutant non-small-cell lung cancer with a cystine-depleting enzyme has been shown to inhibit tumor growth in mice. These findings also suggest that targeting ferroptosis may be a promising therapeutic strategy for EGFR mutant cancer.

Alternatively, we analyzed the regulation of hyper-mutated TP53, EGFR, PIK3CA, RB1, and IDH1 on other ferroptosis proteins. Compared to the EGFR wild-type, ACSL4 mRNA and protein abundances were significantly down-regulated in EGFR-mutated patients (Figure S2). ACSL4 was found to act as a tumor suppressor and promoted ferroptosis [30, 31]. Depletion of ACSL4 has been reported to inhibit ferroptosis in multiple cancer cells including glioma cells [32]. Another example was increased mRNA and protein abundances of FADS2 in IDH1-mutated patients (Fig. 2E). FADS2 could suppress ferroptosis by inhibiting the accumulation of lipid peroxides (LPO) and intracellular iron and promote tumor initiation and development [33].

### Ferroptosis regulators facilitate predicting patients’ survival outcomes

**Fig. 3:**
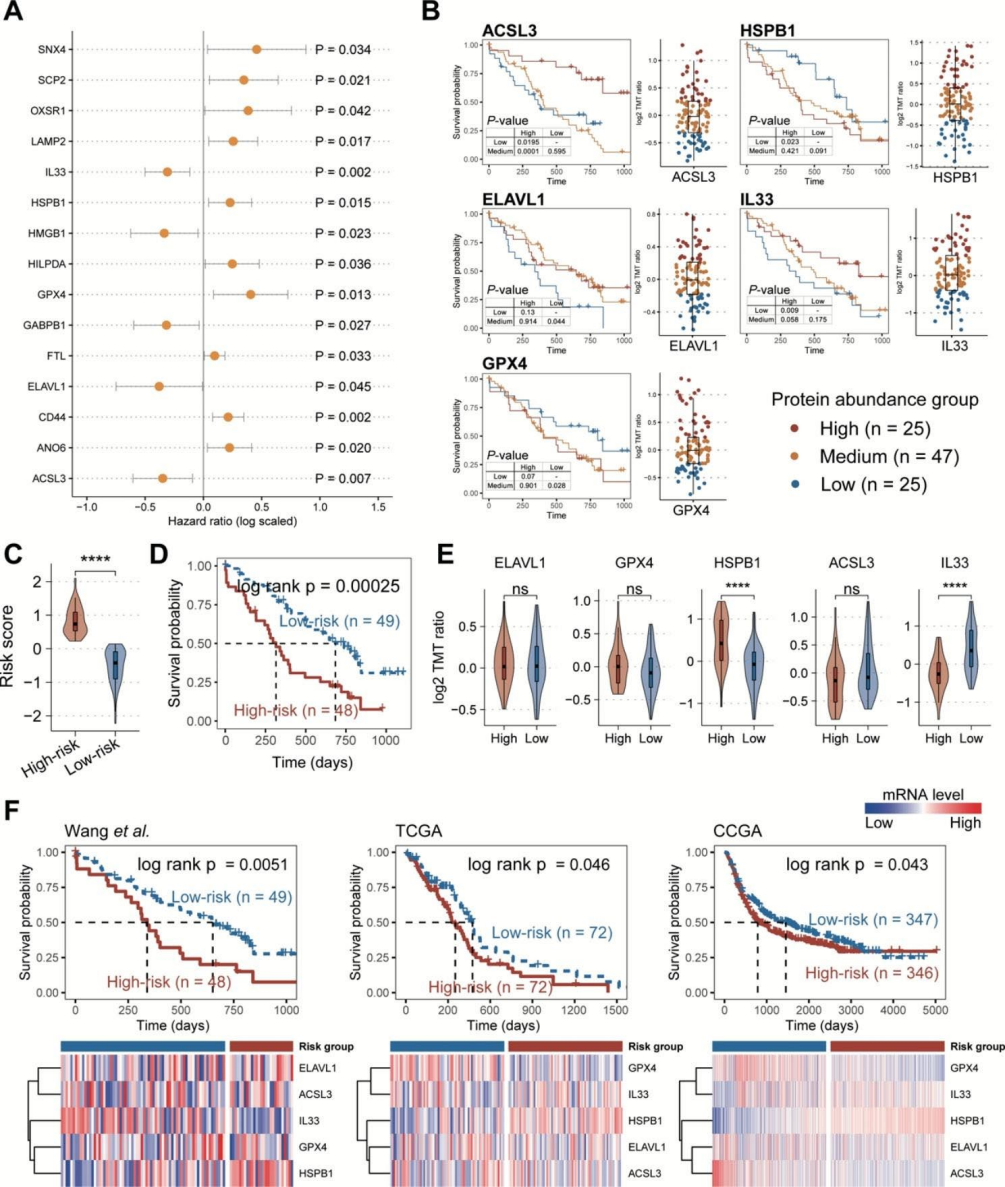
Ferroptosis regulators indicated the prognosis values in GBM. (**A**) Forest plot showing the result of univariate Cox-regression analysis for correlation between the ferroptosis regulators and the overall survival. (**B**) Kaplan-Meier curves of GBM samples stratified by the protein abundances of five risk ferroptosis regulators with log-rank test *P*-value provided, respectively. To determine the protein abundance groups, we divided the corresponding protein expression levels into quartiles. Samples with expression levels falling below the first quartile were assigned to the low abundance group, and those above the three-quarters loci were assigned to the high abundance group. (**C**) Violin plot showing the difference in risk score between high-risk and low-risk groups. (**D**) Kaplan-Meier curve of GBM samples stratified by the risk groups with log-rank test *P*-value provided. (**E**) Violin plots showing the differences in protein abundances of five risk ferroptosis regulators between high-risk and low-risk groups. (**F**) Validation of risk ferroptosis regulators in internal and external RNA-seq cohorts. *P*-values were calculated by Mann–Whitney U test (**C**, **E**)

To better understand the potential clinical implications of ferroptosis proteins in GBM, we explored the prognostic ferroptosis proteins using the univariate Cox regression model (See the “Materials and methods” section). A total of 15 ferroptosis regulators were significantly associated with patients’ overall survival (OS) probability, including ten risk markers and five protective markers (Fig. 3A). Subsequently, we applied the multivariate Cox regression and stepwise regression models to identify a robust ferroptosis prognosis signature (See the “Materials and methods” section). A five-ferroptosis-protein signature (FPS), consisting of ACSL3, HSPB1, ELAVL1, IL33, and GPX4, showed a good performance for predicting patients’ survival outcomes in both training and test sets (Fig. 3B and Figures S3A-B). We also examined the FPS signature using the CPTAC-transcriptome data from the same samples by uni- and multi-variate Cox regression analyses (Figure S4). Notably, there was no significant association between their RNA levels and patients’ OS probability. This evidence also highlights that proteome-based analyses will identify prognostic markers that are distinct from the commonly found at the transcriptomic level, providing additional values to survival indications for tumor patients.

In addition, we employed a risk-scoring model based on the FPS protein abundances and subgrouped patients into high- and low-risk groups (Fig. 3C) (See the “Materials and methods” section). The FPS risk score was observed to have a significant difference between the high- and low-risk groups (Fig. 3D). Furthermore, after adjusting for patients’ age, gender, BMI, and race, the FPS risk score was found to be an independent prognostic indicator of poor prognosis (Figure S3C). We also compared the protein abundances of five FPS proteins between high- and low-risk groups and found that the expression levels of HSPB1 were significantly higher in the high-risk group, whereas IL33 expression levels were significantly higher in the low-risk group (Fig. 3E). In contrast, Considering the lack of GBM MS data, we also validated the efficiency of FPS in internal (corresponding CPTAC GBM-RNA-seq data) and external datasets (TCGA and CCGA GBM-RNA-seq data). In these validation datasets, FPS also showed a good performance for indicating patients’ survival outcomes (Fig. 3F).

### HSPB1 phosphorylation activity is closely associated with high-risk patients

**Fig. 4:**
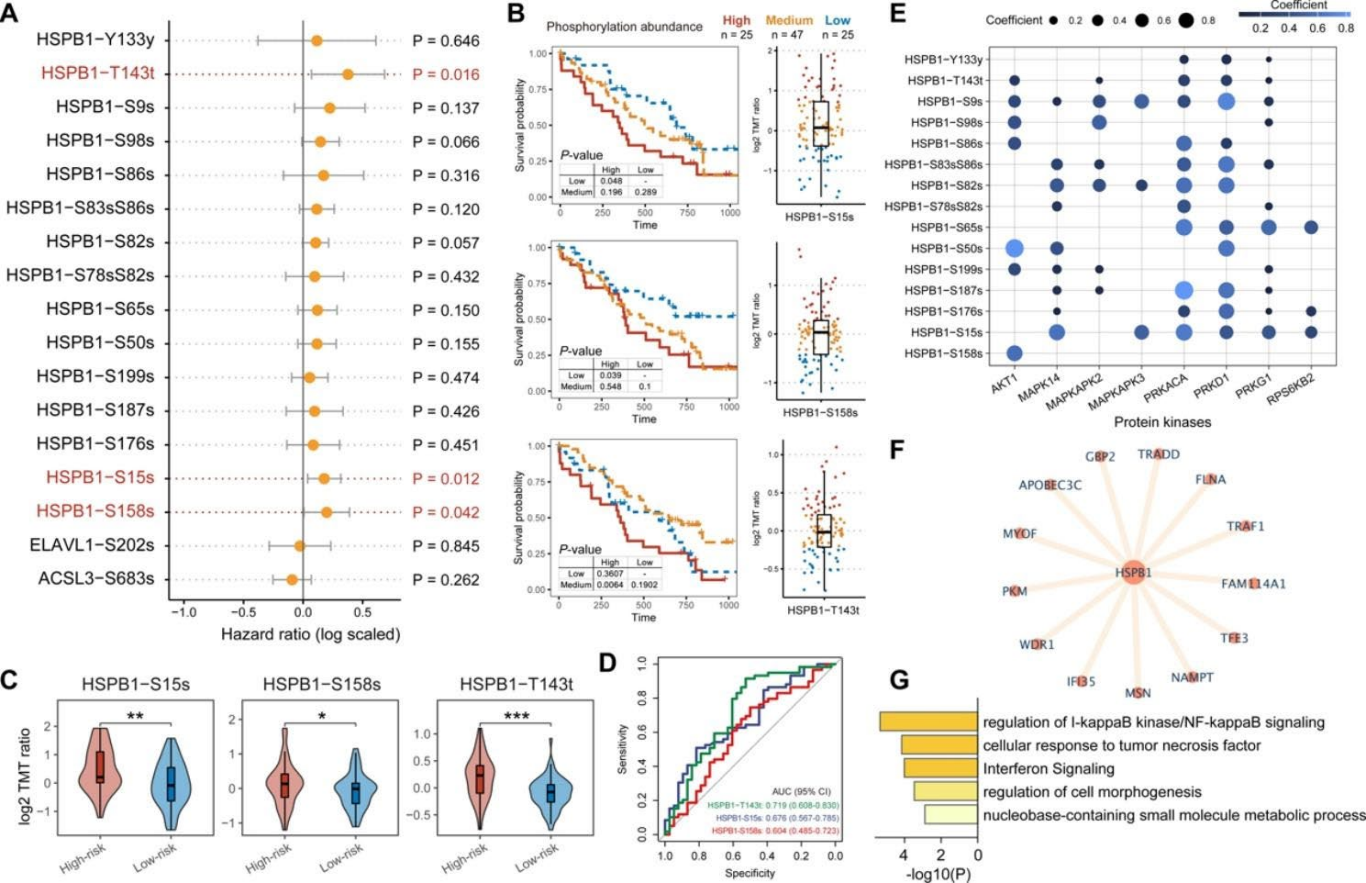
HSPB1 phosphosites contributed to GBM survival. (**A**) Forest plot showing the result of univariate Cox-regression analysis for correlation between the phosphosites of five risk ferroptosis regulators and the overall survival. (**B**) Kaplan-Meier curves of GBM samples stratified by the phosphorylation abundances of HSPB1 with log-rank test P-value provided, respectively. (**C**) Violin plots showing the differences in phosphorylation abundances of HSPB1 between high-risk and low-risk groups. *P*-values were calculated by Mann–Whitney U test as *P ≤ 0.05; **P ≤ 0.01; ****P ≤ 0.0001. (**D**) ROC curve showing the performances of HSPB1 phosphosites for distinguishing GBM risk groups. (**E**) Bubble plot showing the correlations between HSPB1 phosphosites and kinases. (**F**) Co-expressed network of HSPB1. (**G**) Function enrichment of HSPB1 co-expressed proteins using Metascape

For a large subset of proteins, phosphorylation is tightly associated with protein activity and is a key point of protein function regulation [34]. We next explored the role of FPS phosphosites in patients’ survival using the univariate Cox regression. Notably, we found three phosphosites of HSPB1 (HSPB1-S15s, HSPB1-S158s, and HSPB1-T143t) showed a significant association with the patient’s prognosis (Fig. 4A). Patients with high-abundance phosphosites usually had poor survival outcomes (Fig. 4B). We also compared the phosphorylation abundances of these sites between high-risk and low-risk groups. The result showed that the three sites were significantly activated in the high-risk group (Fig. 4C), further highlighting that activated HSPB1 was a poor prognostic marker in human GBM. Alternatively, the three phosphosites performed well in distinguishing between high and low-risk groups, with an area under the ROC curve (AUC) of 0.719 (HSPB1-T143t), 0.676 (HSPB1-S15s), and 0.604 (HSPB1-S158s), respectively (Fig. 4D). In addition, we explored the HSPB1-related kinases and their associations with HSPB1 phosphosites in GBM (See the “Materials and methods” section), including AKT1, MAPK14, MAPKAPK2, MAPKAPK3, PKD1, PRKACA, PRKD1, PRKG1, RPS6KB2, p70S6Kb [20]. We found that AKT1 was a specific kinase for HSPB1-S158s (Fig. 4E). PRKACA, PRKD1, and PRKG1 were related to HSPB1-T143t and HSPB1-S15s phosphorylation (Fig. 4E). In addition, Sun et al. reported that HSPB1 and its increased activity could act as a negative regulator of ferroptotic cancer cell death [35], which was also reasonable for the prognostic value of HSPB1 in GBM (Figure S5). To further investigate the potential biological functions of HSPB1, we identified the co-expressed proteins of HSPB1 (See the “Materials and methods” section). A total of 13 proteins showed significant association with HSPB1 protein abundance (Spearman’s correlation > 0.6, adjusted P-value < 0.01) (Fig. 4F). Further functional annotation analysis suggested these proteins were significantly enriched in multiple immune-related processes, such as regulation of I-kappaB kinase/NF-kappaB signaling cellular response to tumor necrosis factor, and interferon signaling (Fig. 4G). The evidence indicated that HSPB1 might show crosstalk with the immune microenvironment [36].

### HSPB1 showed correlations with monocyte/macrophage infiltration levels

**Fig. 5:**
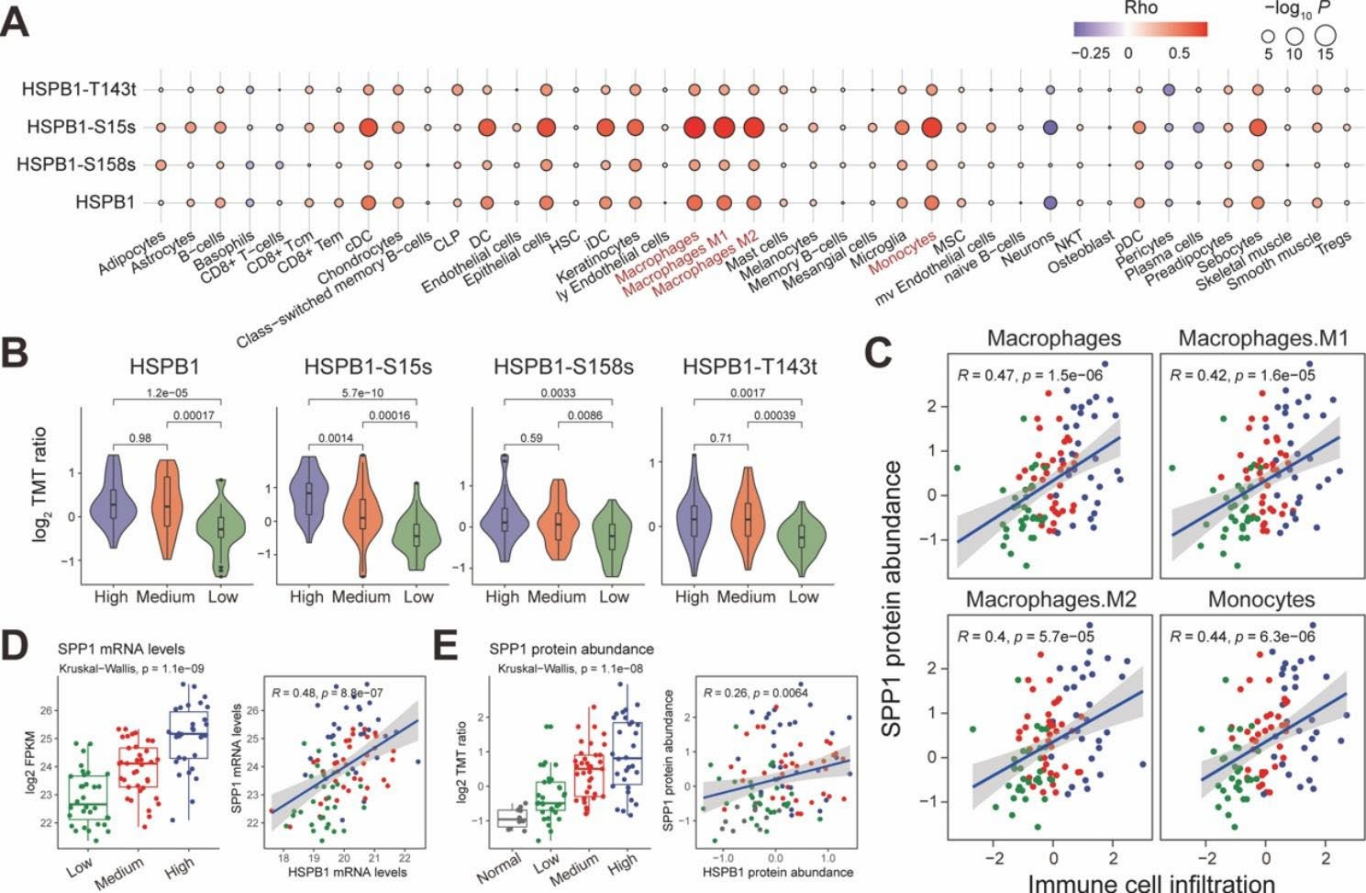
Associations between HSPB1 and tumor microenvironment. (**A**) Bubble plot showing the correlations between immune cell enrichment and protein and phosphorylation abundances of HSPB1. (**B**) Violin plots showing the protein and phosphorylation abundances of HSPB1 among GBM immune subtypes. (**C**) Scatter plot showing the correlation between SPP1 protein abundance and macrophage/monocyte infiltration levels. D-E. Boxplot showing the transcription (**D**) and protein (**E**) levels of SPP1 in different GBM immune subtypes. Scatter plot showing the correlation between SPP1 and HSPB1 protein abundances. The correlation was calculated by Spearman’s correlation test

To better understand the HSPB1’s immunological correlations, we performed the Spearman’s correlation analysis between HSPB1 protein abundance and tumor immune cell infiltration levels. The infiltration levels of 41 immune cells were evaluated by x Cell algorithm [22]. The result of correlation analysis exhibited that HSPB1 was closely associated with monocyte and macrophage infiltrations (Fig. 5A). Subsequently, we identified the GBM immune subtypes (See the “Materials and methods” section). Three immune subtypes were annotated as the high-, medium-, and low- immune levels (Figures S6A-B). Notably, we found patients in high immune subtypes showed poorer prognostic outcomes than the other two subtypes (Figure S6C), which was also consistent with the previous study [37]. We observed the higher protein and phosphorylation abundances of HSPB1 in the immune-high subtype than in immune-medium/low subtypes (Fig. 5C), suggesting the high activity of HSPB1 in the immune-high microenvironment. Notably, monocyte and macrophage also exhibited the high infiltration levels in the immune-high subgroup (Figure S6A). Previous studies have illustrated that tumor-associated macrophages play an important role in tumor maintenance and progression, and in particular, macrophages secrete secreted-phosphoprotein 1 (SPP1) to sustain glioma cell survival [38, 39]. We also observed significant positive correlations between SPP1 protein abundance and macrophage infiltration levels in both CPTAC and TCGA cohorts (Fig. 5C and Figure S7). In addition, SPP1 showed a significantly positive association with HSPB1 at both mRNA and protein levels (Fig. 5D-E). Previous studies demonstrated that SPP1 was able to activate AKT to promote glioma growth [38]. As the above result shows, AKT was also an important kinase of HSPB1 phosphorylation. These results suggested that macrophages secreted SPP1 could be a potential activator for HSPB1, thus inhibiting glioma cell ferroptosis.

### Potential ferroptosis-inducing therapy strategy targeting HSPB1

**Fig. 6:**
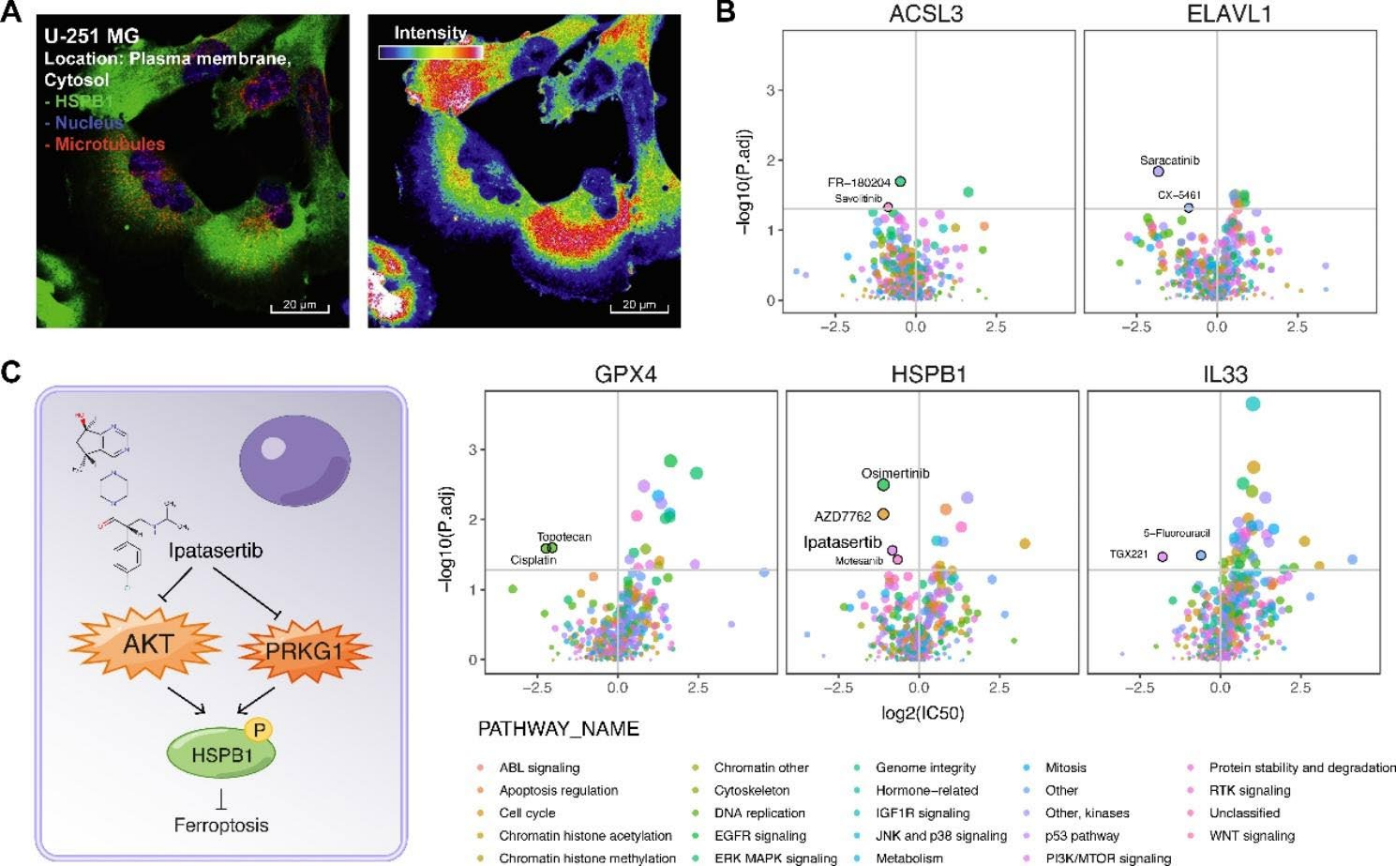
Potential ferroptosis-inducing therapy strategy targeting HSPB1. **A**. Immunofluorescent (IF) staining of HSPB1 in U-251 MG cells. For full IF staining profiles, view the protein at https://www.proteinatlas.org. **B**. Volcano plots showing the differences in drug sensitivity between glioma cells with high mRNA expression of HSPB1 versus remaining glioma cells. *P*-values were calculated by Mann–Whitney U test. **C**. Illustration showing ipatasertib could inhibit kinases AKT and PRKG1 activity, decreasing HSPB1 phosphorylation, thus promoting cancer cell ferroptosis

We next explored the potential ferroptosis-inducing therapy strategy mainly targeting HSPB1 in GBM. Subcellular location showed that HSPB1 mainly exists in the plasma membrane in the U-251 MG glioma cell (Fig. 6A). We analyzed 52 glioma cell lines from Genomics of Drug Sensitivity in Cancer (GDSC) project [24] and their drug sensitivity (See the “Materials and methods” section). Ipatasertib showed a lower IC50 value in high-HSPB1 glioma cells than in low-HSPB1 glioma cells (Fig. 6B). And ipatasertib could act on PI3K/mTOR signaling in glioma cells. Previous studies also showed that ipatasertib was a novel highly selective ATP-competitive pan-Akt inhibitor, showing a strong antitumor effect in a variety of cancer [40]. We also investigated the drug-gene interaction from DGIdb [41]. We found two kinases of HSPB1, AKT and PRKG1 could interact with ipatasertib (Fig. 6C). A previous study has demonstrated that the knockdown of HSPB1 could enhance erastin-induced ferroptosis (erastin is a specific ferroptosis-inducing compound) [35]. The evidence showed a potential ferroptosis-inducing therapy strategy that ipatasertib could inhibit HSPB1 phosphorylation, enhancing erastin-induced ferroptosis, and thus killing tumor cells.

## Discussion

Exploratory genome and transcriptome analyses of clinical cancer cohorts have demonstrated the value of a systems-level understanding of human cancers [42, 43]. However, few studies have focused on the proteome. With the improvement of mass spectrometry (MS), we can measure the actual druggable targets at proteome levels. In this study, we systemically characterized the dysregulated ferroptosis proteins and their potential clinical utilizations. Ferroptosis was a comprise process caused by multiple biological factors. Hence, some ferroptosis regulators also played the important role in other cellular processes. For example, p53 is an important tumor suppressor gene that regulates cell cycle arrest and apoptosis [44, 45]. While unlike apoptotic cell death, p53 activation alone is not sufficient to induce ferroptosis directly, it is able to modulate the ferroptosis response in the presence of ferroptosis inducers such as GPX4 inhibitors or high levels of ROS [45]. By analyzing the effect of genomic alterations on the protein abundances of ferroptosis regulators, we found that p53 mutation can upregulate its expression levels. However, the role of p53 remains a debate as an inducer or inhibitor of ferroptosis [46]. Nevertheless, consistent with previous studies [27–29], we also observed the inhibited ferroptosis activity in GBM tissues. Decreased ferroptosis may be an important reason why tumor cells are able to escape programmed cell death. In our future studies, the experimental methods will also be used for further validation. In addition, we found that in EGFR-mutant samples, the protein abundances of ACSL4 were significantly downregulated than the wild-type patients. ACSL4 has been reported could act as an essential component for ferroptosis execution and dictate ferroptosis sensitivity by shaping cellular lipid composition [11]. In vitro models, researchers found that transcription factor SP1 could bind directly to the ACSL4 promoter region to increase its expression [47]. Remarkably, SP1 was also a downstream protein of EGFR/p38-MAPK signaling, whose phosphorylation and activation were closely correlated to EGFR [48, 49], implying that EGFR mutant could regulate ACSL4 expression by modulating SP1 phosphorylation and activation.

In addition, immune subtype analysis reveals that GBM with low immunity has a better overall survival rate than tumors with high immunity, which was different from other cancer types, such as non-small cell lung cancer and cutaneous melanoma [37, 50–52]. Feng et al. found that GBM with high immunity had higher tumor genomic instability and tumor stemness, which might cause this phenomenon [37]. Another potential explanation is that the inflammatory tumor microenvironment promotes the progression and exacerbation of gliomas [53]. In this study, we found that HSPB1 was highly expressed and showed high phosphorylation levels in the high immune subtypes. Besides, HSPB1 and its phosphosites were correlated with infiltration levels of macrophages. Highly-infiltrated macrophages had also been demonstrated as a poor prognostic biomarker in GBM [38]. These results showed that the linkage between macrophages and HSPB1 could play an important role in GBM prognosis.

*SPP1*^+^ macrophages played a complex role in multiple cancers. In colorectal cancer, patients with high *SPP1* expression achieved less therapeutic benefit from an anti-PD-L1 therapy cohort [54]. In GBM, researchers also reported that macrophage-secreted SPP1 could activate AKT expression [38]. AKT, as a kinase of HSPB1, could regulate HSPB1 phosphorylation activity. The evidence also suggested that macrophage-secreted SPP1 could be an activator of HSPB1 in glioma cells and indirectly inhibit tumor cell ferroptosis. The result also provides a future direction in the exploration of crosstalk between tumor microenvironments.

Finally, we explored a potential ferroptosis-inducing therapy strategy. We found that ipatasertib could inhibit AKT and PRKG1 activity. And AKT and PRKG1 were also kinases of HSPB1, which indicated that ipatasertib could be an indirect inhibitor of HSPB1 to enhance glioma ferroptosis. Sun et al. reported knockdown of HSPB1 could enhance erastin-induced ferroptosis [35]. These results also prompt a potential combination drug strategy, i.e., ipatasertib-erastin inducing ferroptosis of glioma cells.

## Electronic supplementary material

Below is the link to the electronic supplementary material.

Supplementary Material 1

## Data Availability

The datasets generated and/or analysed during the current study are available in the CPTAC (http://linkedomics.org/data_download/CPTAC-GBM/), TCGA (https://portal.gdc.cancer.gov/), and CCGA (http://www.cgga.org.cn/).

## Abbreviations

TCGA: The Cancer Genome Atlas
CGGA: Chinese Glioma Genome Atlas
FPF: ferroptosis-protein signature
ROC: Receiver operating characteristic curve
AUC: Area under the ROC curve.
