# Supplementary material for: Proteogenomic characterization of ferroptosis regulators reveals therapeutic potential in glioblastoma
Source: BMC Cancer. 2023 May 8;23:415. doi: 10.1186/s12885-023-10894-3 (PMC10165763; doi:10.1186/s12885-023-10894-3)
Supplement: Supplementary file 1 — Supplementary Material 1 [file 12885_2023_10894_MOESM1_ESM.pdf]

Proteogenomic Characterization of Ferroptosis Regulators Reveals Therapeutic Potential in Glioblastoma

Wang *et al.*

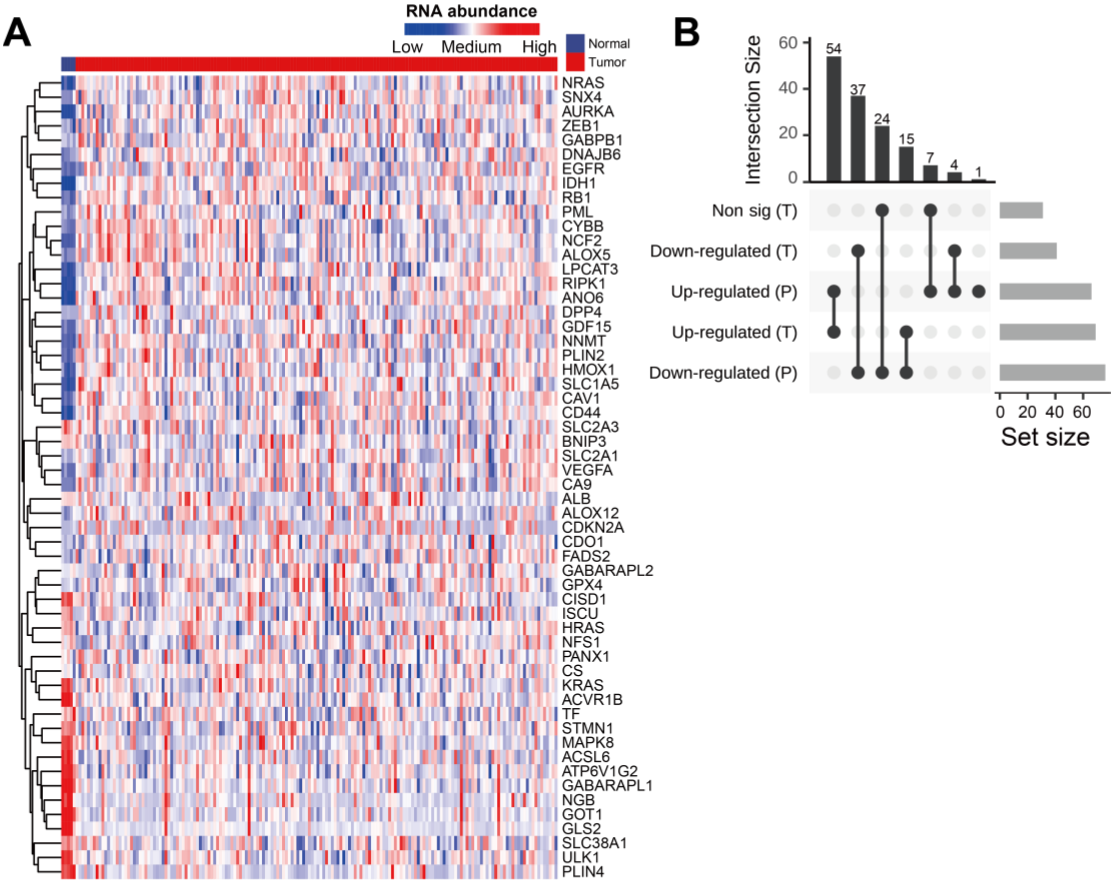

Figure S1. Differentially expressed ferroptosis regulators in TCGA-GBM cohort. A. Heatmap showing the RNA expression levels of the differentially expressed ferroptosis regulators (as shown in Figure 1C proteins) between GBM tumor tissues and normal brain tissues in the TCGA-GBM cohort. B. Upset plot showing the overlap between differentially expressed proteins [Up-regulated (P) and Down-regulated (P)] from the CPTAC dataset and differentially transcribed genes [Up-regulated (T), Down-regulated (T), and Non-sig (T)] from the TCGA dataset.

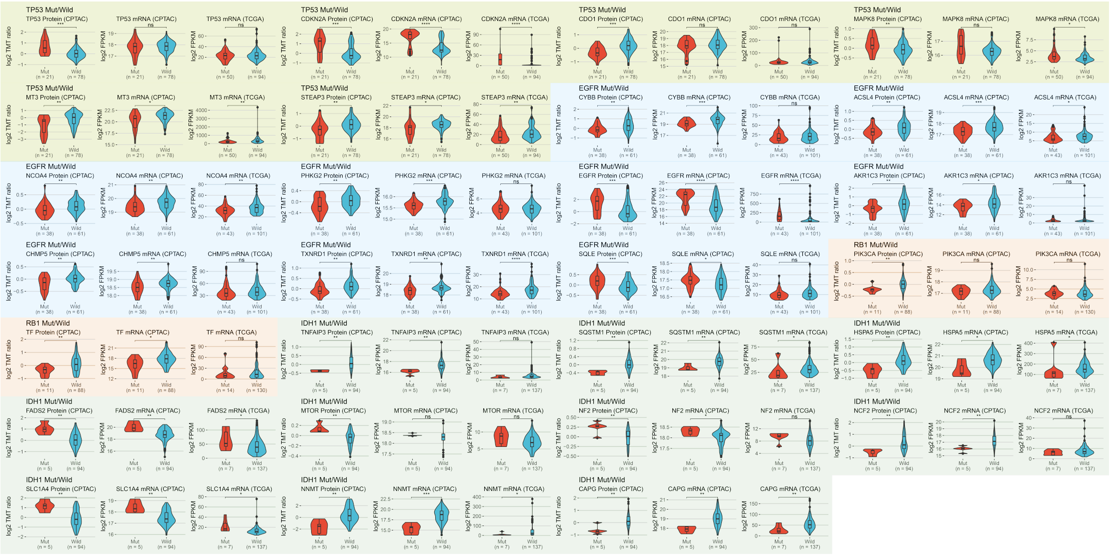

Figure S2. The effects of key mutations on ferroptosis regulators in CPTAC-GBM cohort. In each panel, violin plot showing the expression levels between mutated and wild samples using the CPTAC-GBM proteome dataset (left), CPTAC-GBM transcriptome dataset (middle), and TCGA-GBM transcriptome dataset (right). *P*-values were calculated by Mann–Whitney U test as $^{*}P \leq 0.05$; $^{**}P \leq 0.01$; $^{****}P \leq 0.0001$.

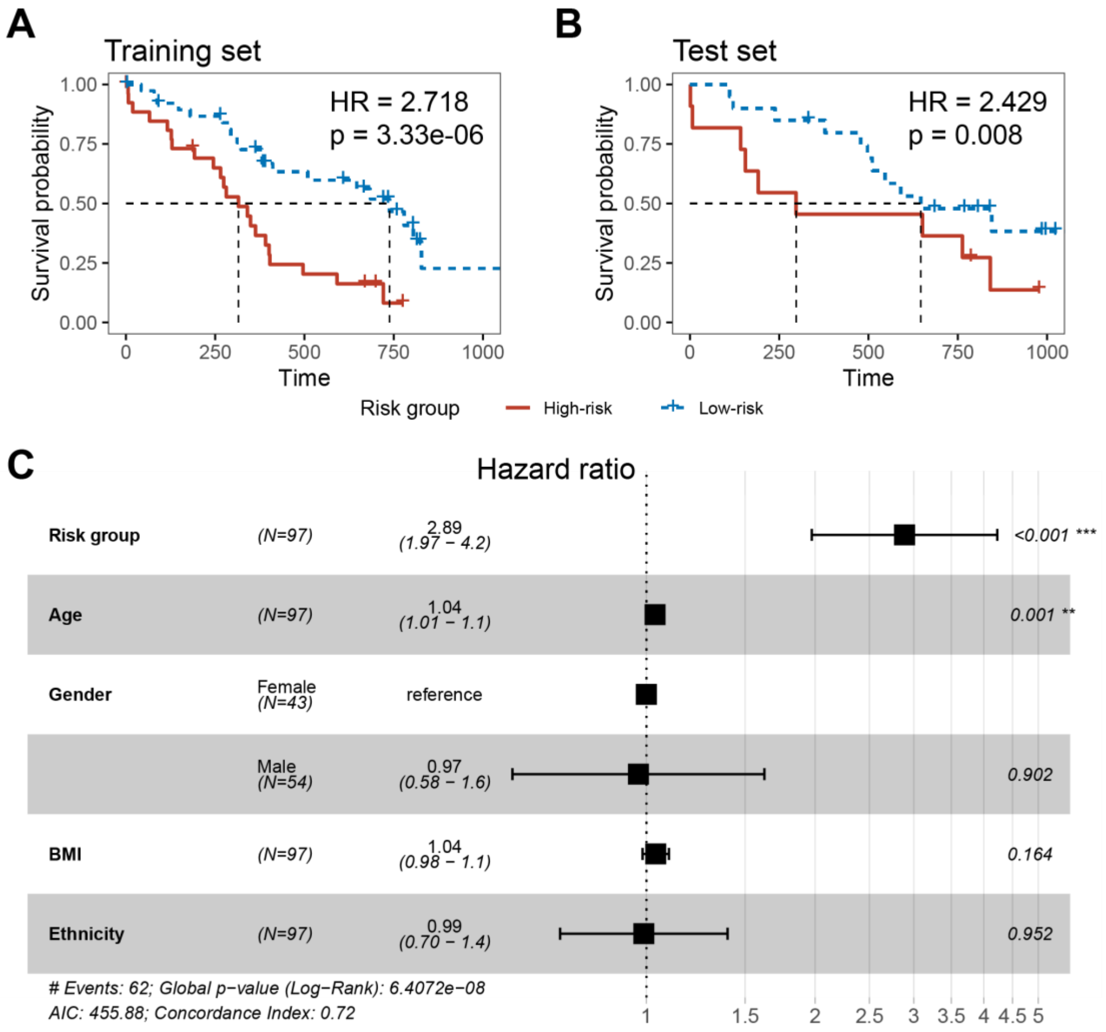

Figure S3. FPS signature in CPTAC proteome dataset. A-B. Kaplan-Meier curve of GBM samples stratified by the risk groups with log-rank test P-value provided in the training set (A) and test set (B). C. Forest plot showing the result of multivariate Cox-regression analysis for correlation between the FPS risk groups and clinical features and the overall survival.

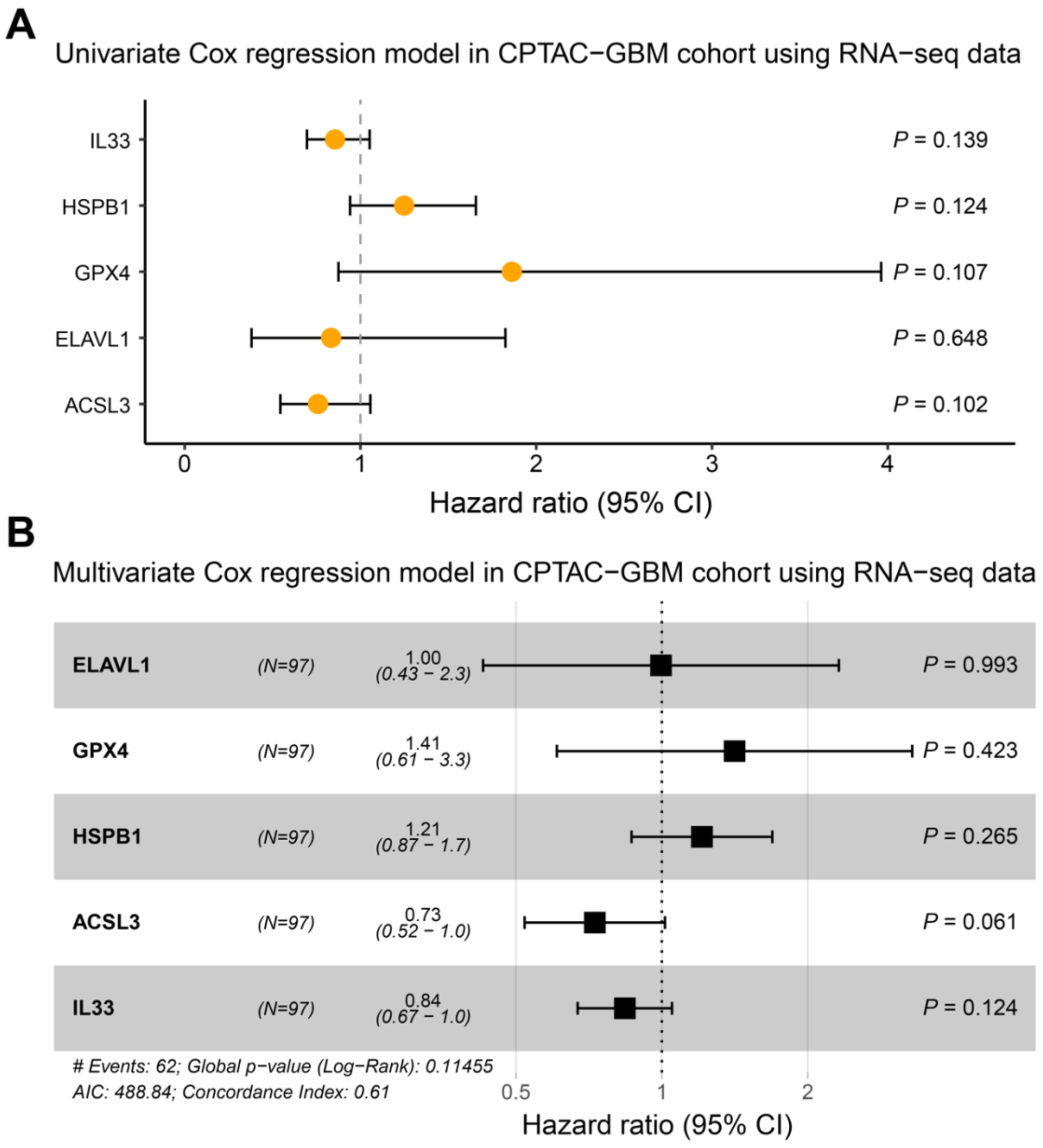

Figure S4. FPS signature in CPTAC transcriptome dataset. A. Forest plot showing the result of univariate Cox-regression analysis for correlation between the transcription levels of ferroptosis regulators in FPS signature and the overall survival. B. Forest plot showing the result of multivariate Cox-regression analysis of the factors associated with overall survival.

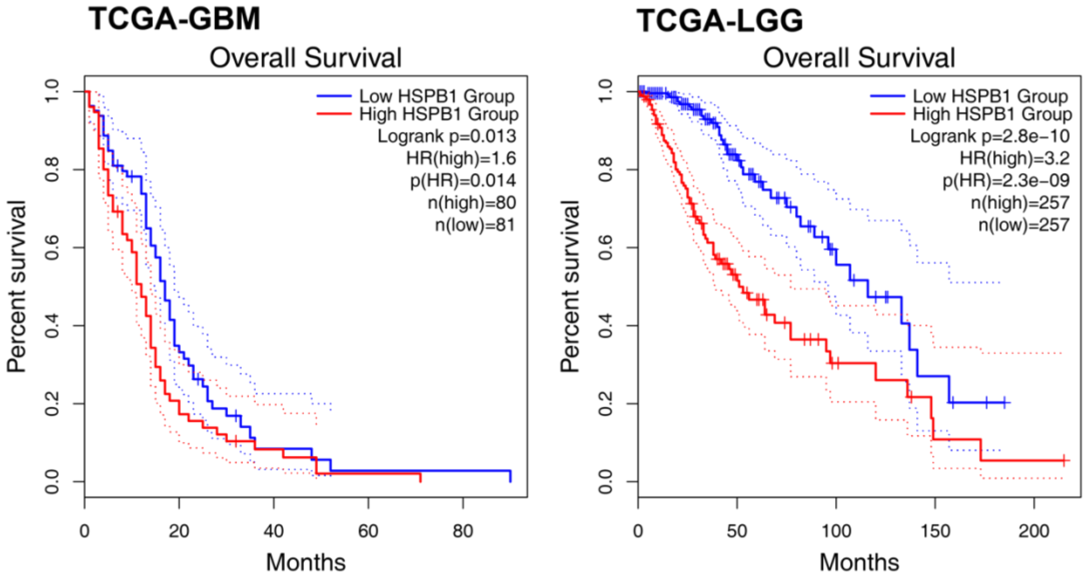

Figure S5. Kaplan-Meier curve of samples stratified by the HSPB1 mRNA levels with log-rank test P-value provided in TCGA-GBM and TCGA-LGG cohorts.

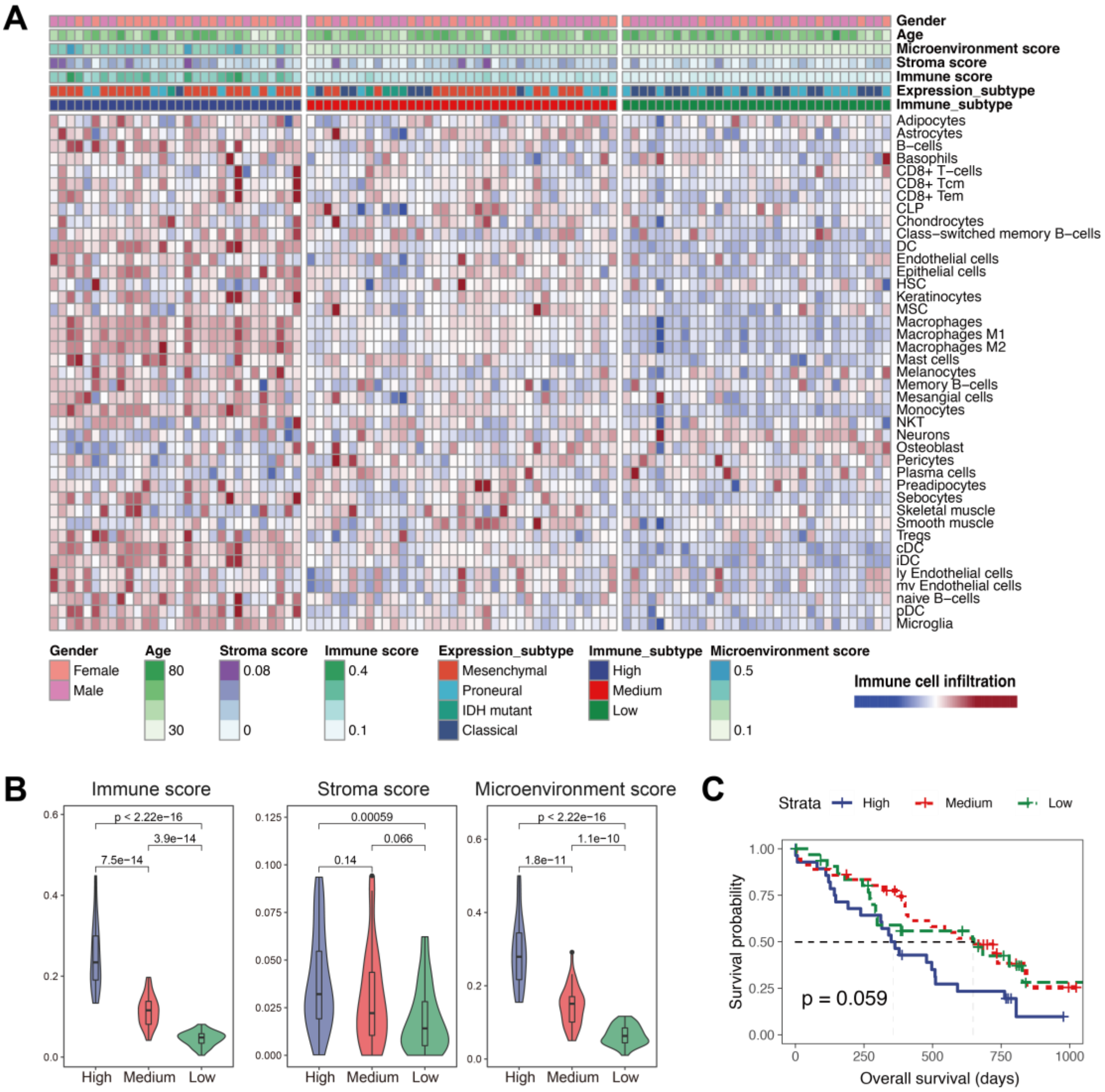

Figure S6. Immune subtypes of CPTAC-GBM cohort. A. Heatmap showing the enrichment scores of immune cells in GBM immune subtypes (High, Medium, and Low). B. Violin plots showing the immune, stroma, and microenvironment scores calculated by xCell among GBM immune subtypes. C. Kaplan-Meier curve of GBM samples stratified by the immune subtypes with log-rank test *P*-value provided in CPTAC proteome cohort.

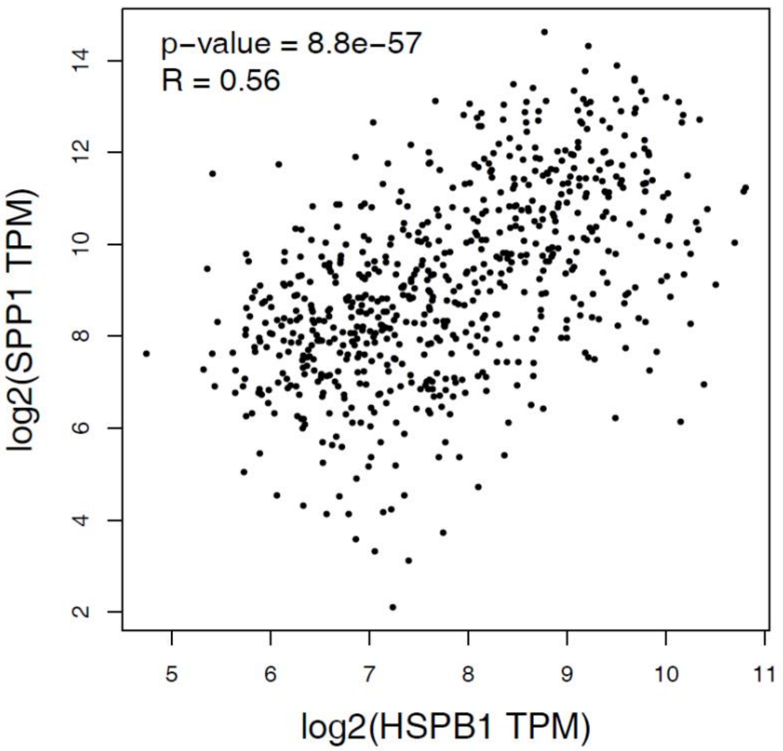

Figure S7. Scatterplot showing the correlation between SPP1 mRNA and HSPB1 mRNA in TCGA-GBM and TCGA-LGG cohorts. The correlation was calculated by Spearman's correlation test.
